# Associations of circulating vitamins with 10-year retinal neurodegeneration: the Alienor Study

**DOI:** 10.1016/j.jnha.2024.100286

**Published:** 2024-06-10

**Authors:** Bénédicte MJ Merle, Cédric Schweitzer, Marie-Bénédicte Rougier, Audrey Cougnard-Grégoire, Laure Gayraud, Marie-Noëlle Delyfer, Jean-François Korobelnik, Cécile Delcourt

**Affiliations:** a Univ. Bordeaux, INSERM, BPH, U1219, Bordeaux, France; b CHU de Bordeaux, Service d’Ophtalmologie, Bordeaux, France

**Keywords:** Vitamins, Nutrition, Neurodegeneration, Retina, Retinal nerve fiber layer (RNFL), Cohort

## Abstract

**Objective:**

To investigate the associations between circulating vitamins A, D, E, B6, B9, B12 and longitudinal changes in retinal nerve fiber layer (RNFL) thickness.

**Methods:**

The Alienor study, a prospective population-based cohort (Bordeaux, France), includes 963 individuals aged 73 years or older at baseline. The present study included 646 participants with complete RNFL measurement and vitamins. Study period is from 2009 to 2020. Peripapillary RNFL thickness was measured using spectral domain optical coherence tomography (SD-OCT). Plasma vitamins A, D and E and, serum vitamins B6, B9 and B12 were measured from blood sample. We performed linear mixed models, adjusted for age, gender, axial length, family history of glaucoma, and alcohol consumption to evaluated associations between vitamins and RNFL thickness changes over time.

**Results:**

Individuals having higher concentrations of vitamin E, D and B9 had a slower RNFL thinning during the 10-years of follow-up. Indeed, a 1-standard deviation (SD) increase of vitamin E (10.8 μmol/L), D (17.6 nmol/L) and B9 (11 μmol/L) were associated with slower RNFL thinning by 0.14 μm/year (95% confidence interval (CI), 0.03−0.25, p = 0.01), 0.14 μm/year (95% CI, 0.02−0.27, p = 0.02) and 0.11 μm/year (95% CI: 0.007−0.21, p = 0.04), respectively. No significant associations were observed for vitamins A, B6 and B12 with RNFL thinning.

**Conclusions:**

Higher levels of vitamins E, D and B9 were associated with a slower RNFL thickness on SD-OCT over time, suggesting that those vitamins may contribute to the neuroprotection of the retina.

## Highlights

• Subjects with higher circulating vitamin E, D and B9 had a slower RNFL thinning over time.
• No significant associations were observed for vitamins A, B6 and B12 with RNFL thinning.

## 1 Introduction

Glaucoma is the second leading cause of blindness worldwide and its prevalence is estimated up to 111.8 million in 2040 [1]. This progressive degenerative optic neuropathy is characterized by the irreversible and progressive death of retinal ganglion cells. Intraocular pressure (IOP) remains the main modifiable risk factor recognized in the development and progression of glaucoma [[2], [3], [4]], but studies have shown that lifestyle factors, such as smoking and the lack of physical exercise, are also associated with the development of the disease [5]. In conjunction with current treatments to lower IOP, neuroprotection is a key concept in glaucoma prevention and stabilization, aimed at preserving the structure and function of retinal ganglion cells. In fact, retinal nerve cells have a very high level of continuous metabolic activity and a highly developed energy cycle, enabling them to transmit visual stimuli from the retinal photoreceptors to the brain [6]. The neuroprotective and anti-inflammatory properties of certain nutrients could therefore play an important role in this context. Complementary to treatment, the nutritional approach in retinal neurodegeneration pathologies could be of importance to help reducing associated burden.

The associations between nutrition and retinal neurodegeneration have not yet been fully explored. Higher intake of certain food such as tea [7], fruits and vegetables [8,9], have been linked to lower IOP levels and lower incidence of glaucoma. Those foods are a major sources of vitamins A, B and E. High dietary intake of vitamin A may have a beneficial effect on glaucoma [10], as well as, a high ACE-vitamin index, a combination of vitamin A, C and E [11]. Adhering to a healthy diet was associated with a lower prevalence [12] and incidence [13] of glaucoma. However, dietary data rely to the participants’ memory and do not take into account bioavailability; nutritional biomarkers are thus more objective to assess nutritional exposure. Several studies have explored the associations between circulating vitamins and glaucoma. Lower circulating level of vitamin A (retinol) [14], B3 (nicotinamide) [15], B6 [16,17], B9 (folate) [16,18] and B12 [16] have been reported in glaucoma patients. Longitudinal data on the protective effect of vitamins against retinal neurodegeneration are sparse. Oxidative stress plays an important role in retinal ganglion cells (RGC) death in glaucoma, thus antioxidant properties of vitamins E and B9 are also important to consider. Moreover, vitamins D, B6, B9 and B12 have neuroprotective and anti-inflammatory effects, and could therefore have a key role in retinal neurodegeneration [19].

RNFL thinning is a well-established OCT biomarker for early detection [20,21] and progression of retinal neurodegeneration including glaucoma^15^. Given the evidence for vitamins as neuroprotective and antioxidant agents against retinal neurodegeneration, the aim of our study is to explore the longitudinal associations between circulating vitamins A, D, E, B6, B9 and B12 with RNFL thinning in a population-based cohort of French older adults.

## 2 Methods

### 2.1 Study aims

The Alienor Study (Antioxydants, Lipides Essentiels, Nutrition et maladies OculaiRes) is an ongoing prospective population-based study (www.alienor-study.com) aiming at assessing the associations of age‐related eye diseases with nutritional factors and other major determinants of eye disease. [22]

### 2.2 Study sample

**Fig. 1:**
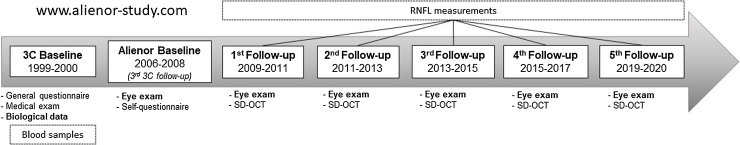
Alienor Study data collection.

Participants were recruited from the Three-City (3C) Study which included 9 294 subjects from electoral lists aged 65 years or more from three French Cities (Bordeaux, Dijon and Montpellier) [23]. Three-City participants were recruited in 1999–2001 and followed-up every two years since. The Alienor Study consists of eye examinations, which are offered to all participants of the 3C cohort in Bordeaux since the 3C third follow-up (2006–2008) and every two years as shown in Fig. 1. Since 2009, technological innovations in ophthalmological imaging made it possible to add a spectral-domain optical coherence tomography (OCT) examination to the eye check-up, enabling the measurement of RNFL thickness. These measurements are available at five eye examinations from 2009 to 2020 (Fig. 1). This research was approved by the Ethical committee of Bordeaux (Comité de Protection des Personnes Sud-Ouest et Outre-Mer III) in May 2006. All participants provided written informed consent in accordance with the Declaration of Helsinki to participate in the study.

### 2.3 Retinal nerve fiber layer thickness measurements

At each visit, participants underwent a full eye examination at the Bordeaux University Hospital, including visual acuity, refraction, intraocular pressure, retinal photographs and OCT examination [22]. Spectral-domain OCT examinations were performed using Spectralis® (software version 5.4.7.0, Heidelberg Engineering, Heidelberg, Germany). The same machine was used for all participants at all follow-up visits. Peripapillary RNFL thickness acquisitions were obtained using the high-resolution protocol and calculated using the 3.45 mm circle scan diameter centered on the optic disc [21]. Global peripapillary RNFL thickness was automatically calculated by the device. Correction for fovea-disc orientation (FoDi) is incorporated in the software and a real-time eye tracking system is used to compensate for eye movements. All images were acquired and reviewed by specially trained technicians to control quality of signal strength [(> 15 dB) (Range: 0–40)], accurate centration and segmentation of the peripapillary RNFL thickness acquisition. In case of an imprecise segmentation of inner and outer boundaries of the peripapillary RNFL on the raw image, manual corrections were performed by a trained technician masked to clinical data. Signal strength lower than 15 dB or acquisitions with artefacts, including staphyloma or atrophy on the circle scan, were excluded from the analysis.

### 2.4 Vitamins measurements

Vitamins measurements were determined from fasting blood samples collected at the 3C baseline visit into heparinized evacuated tubes and centrifuged at 1000× *g* for 15 min and stored at −80 °C until determinations. None of the people involved in the determination had any accesses to ocular clinical findings at any time of the study.

Plasma retinol (vitamin A) and alpha-tocopherol (vitamin E) concentrations were determined by high-performance liquid chromatography (HPLC) [24].

Serum pyridoxine (vitamin B6) concentrations (nmol/L) were measured with liquid chromatography coupled to tandem mass spectrometry at CERBA laboratory (Saint Ouen l’Aumône, France). Serum folate (vitamin B9, nmol/L) and serum cobalamin (vitamin B12, pmol/L) concentrations were measured with chemiluminescence immunoassay (Abbott Architect i2000SR) at EXALAB laboratory (Le Haillan, France).

Plasma 25(OH)D (vitamin D) concentrations were assessed with a one-step immunoassay (Architect 25-OH Vitamin D Assay; Abbott Diagnostics, Germany) as described elsewhere [25]. We used a “deseasonalized” plasma 25(OH)D concentration variable.

We first regressed the measured 25(OH)D concentrations (in nmol/L) on calendar time using the following periodic function:

$$y_{t}=\;\beta_{0}+\beta_{1}\;\text{sin}\left(\frac{2\pi t}{365}\right)+\;\beta 2\;\text{cos}\left(\frac{2\pi t}{365}\right)$$

where *y_t_* denotes measured plasma 25(OH)D concentration, *t* denotes the day of the year the sample was collected, and β*j* (j = 0, 1, 2) are estimated regression coefficients; we then extracted the residuals from this model (which represent the differences between each individual’s actual 25(OH)D concentration and the concentration predicted by calendar time). Because residuals, by definition, have a mean of zero and negative and positive values, a constant can be added to every value to convey the sense of an actual concentration value; we thus added the residuals of this regression model to the seasonal average to create a deseasonalized vitamin D concentration for each individual. This provides a way to adjust for the seasonal variation of 25(OH)D given that blood samples were collected throughout the year. “January 1” deseasonalized values were arbitrarily chosen for analysis, although in a periodic function any date would be expected to have been equally informative (and subject to the same limitations). The consideration of deseasonalized values was an a priori decision; this computation being largely used in the field of multiple sclerosis [26,27] and dementia [28].

### 2.5 Covariates

Demographic (age, gender), lifestyle (education, income, smoking, alcohol consumption and body mass index (BMI) (weight (kg)/height (m)²)) and medical (cardiovascular diseases, diabetes, hypercholesterolemia and hypertension) data were collected during face-to-face interviews using standardized questionnaire administered by a trained psychologist or nurse at the fifth 3C follow-up visit (2009–2011), corresponding to the first eye examination with RNFL thickness measurement. Hypertension was defined as systolic blood pressure≥140 mm Hg and/or diastolic blood pressure /≥90 mm Hg and/or use of antihypertensive drugs. Diabetes was defined as fasting blood glucose ≥7.0 mmol/L or non-fasting blood glucose ≥11 mmol/L or use of antidiabetic medication or self-reported diabetes. Cardiovascular disease was defined as self-reported myocardial infarct or coronary surgery or coronary or angioplasty or stroke) and hypercholesterolemia (self-reported or treated).

Ophthalmological covariates were collected during the Alienor examinations. Axial length (in mm) was measured using non-contact partial coherence laser interferometry (IOL Master, Carl Zeiss Meditec AG, Jena, Germany).

### 2.6 Statistical analyses

We first compared included to non-included participants regarding demographic, lifestyle, medical, ophthalmological and nutritional data using Wilcoxon rank sum or Pearson's Chi-squared test. To correct for multiple testing, we calculated Benjamini-Hochberg corrected p-values.

The longitudinal relationship between vitamin concentrations and RNFL thickness was studied using linear mixed models with interaction between vitamins concentrations and time. Random effects on the intercept and on the slope of individuals were included in the model, to account for inter-subject variability while taking into account intra-subject and intra-eye correlations (repeated measurements), as previously described [29].

All conditions for the application of the linear mixed model were verified. The linearity of the quantitative variables was investigated using penalized splines with four degrees of freedom and no evidence of departure from linearity was observed for quantitative variables.

Two models were selected based on potential confounders identified in literature. Model 1 was adjusted for age (years) and gender. For logistic and technical reasons, axial length (mm) and family history of glaucoma, both variables strongly associated with the RNFL thickness, were available only in about 60% of the sample. These two variables were integrated in a second model including age, gender and alcohol consumption (for vitamins B). In order to check the robustness of the results, we performed a third model adjusted for age, sex, diabetes, hypercholesterolemia, hypertension and cardiovascular diseases. As alcohol consumption can modify vitamin B status [30,31] as well as RNFL thickness [32], models including vitamins B were further adjusted for alcohol consumption.

Each vitamin was analyzed in separate models. As glaucoma is associated with a thinner RNFL, in sensitivity analyses, we excluded participants with glaucoma at baseline. In our sample few participants were vitamin D supplement users (n = 32), to assess whether the association could be due to supplementation we performed sensitivity analyses by excluding participants declaring taking vitamin D supplement.

All statistical analyses were performed using R, version 3.6.1 (R Core Team). Linear mixed-effects models were performed using the *lmer* function of the *lme4* R package [33].

## 3 Results

### 3.1 Descriptive analysis

**Fig. 2:**
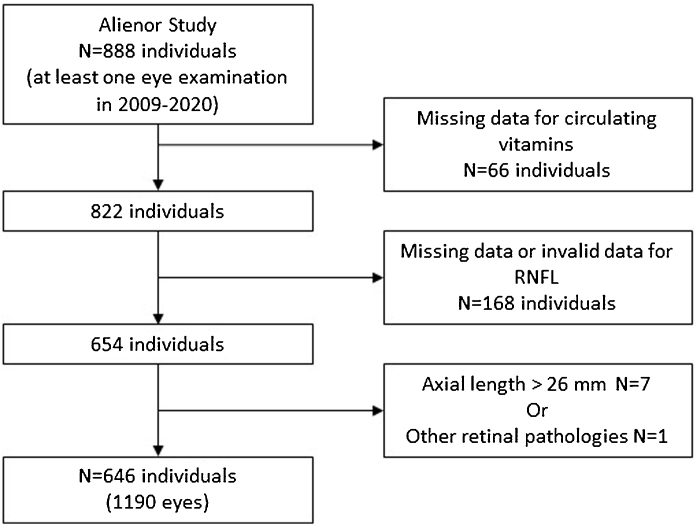
Participants’ selection. Alienor study 2009–2020.

Between the first (2009–2011) and the fifth follow-up visit (2019–2020), 888 individuals had at least one eye examination over the 2009–2020 period. Individuals with missing data for circulating vitamins (n = 66), RNFL (n = 168), with axial length more than 26 mm (n = 7) or having other retinal pathologies (n = 1) were excluded from analyses. The study sample include 646 individuals (1190 eyes with at least one valid RNFL thickness measurement) (Fig. 2).

The mean age at baseline was 82.3 years (standard deviation (SD) = 4.2) and 62% of the study sample were women (Table 1). Two third of the sample had an income of more than 1 500 euros/month and were never smokers. The most common comorbidities were hypertension (84%), hypercholesterolemia (48%), obesity (15%) and diabetes (12%). The mean RNFL thickness was 90.2 μm (SD = 14.0) for each eye.

**Table 1:** Baseline characteristics among included and non-included participants, Alienor study. 2009–2020.

Characteristics	No. (%)		
	Included Participants (n = 646)	Non included Participants (n = 242)	Corrected *P* value^a^
Age, mean (SD), y	82.3 (4.2)	85.5 (5.2)	0.009
Sex	N = 646	n = 240	0.009
Male	248 (38)	61 (26)	
Female	398 (62)	179 (75)	
Education	N = 646	n = 240	0.09
<high school	357 (55)	152 (63)	
≥high school	289 (45)	88 (37)	
Income, euros/month	n = 572	n = 173	0.24
<1,500	192 (32)	70 (40)	
≥1,500	379 (64)	96 (55)	
No answer	21 (4)	7 (5)	
Smoking, pack-years	n = 641	n = 234	0.06
Never smoker	411 (64)	174 (74)	
<20	116 (18)	30 (13)	
≥20	114 (18)	30 (13)	
Alcohol consumption, mean (SD), g/day	n = 600	n = 130	0.42
	12.0 (14.7)	10.4 (12.0)	
Vitamin D supplementation, yes	N = 646	n = 240	0.85
	32 (5)	11 (5)	
Medical conditions			
Body mass index, kg/m²	n = 616	n = 194	0.36
<25	292 (47)	106 (55)	
[25–30]	231 (38)	63 (32)	
>30	93 (15)	25 (13)	
Cardiovascular diseases, yes	N = 646	n = 242	0.03
	22 (3)	19 (8)	
Diabetes, yes	n = 626	n = 213	0.60
	77 (12)	30 (14)	
Hypercholesterolemia, yes	n = 621	n = 210	0.42
	296 (48)	91 (43)	
Hypertension, yes	n = 618	n = 213	0.04
	521 (84)	195 (92)	
RNFL measurements	N = 646		–
Right eye, mean (SD), μm	90.2 (14.0)	–	
Left eye, mean (SD), μm	90.2 (14.2)	–	
Circulating vitamins			
Vitamin A, μmol/L	n = 556	n = 147	0.68
Mean (SD)	1.96 (0.60)	2.03 (0.74)	
Median (IQR) [Min-max]	1.89 (0.67) [0.35−4.76]	1.96 (0.84) [0.70−6.79]	
Vitamin E, μmol/L	n = 556	n = 147	0.60
Mean (SD)	36.1 (10.8)	36.9 (11.3)	
Median (IQR) [Min-max]	35.9 (13.5) [5.5−83.9]	35.7 (14.0) [12.1−73.7]	
Vitamin D^b^, nmol/L	n = 536	n = 73	0.10
Mean (SD)	38.6 (17.6)	34.5 (13.8)	
Median (IQR) [Min-max]	35.2 (17.3) [5.9−252.2]	31.5 (14.7) [9.9−83.7]	
Vitamin B6, nmol/L	n = 601	n = 162	0.42
Mean (SD)	43.4 (38.4)	39.1 (30.2)	
Median (IQR) [Min-max]	34.0 (24.0) [6.0−376.0]	33.0 (21.5) [8.0−235.0]	
Vitamin B9, nmol/L	n = 604	n = 166	0.90
Mean (SD)	19.3 (11.0)	20.1 (13.4)	
Median (IQR) [Min-max]	17.0 (11.0) [4.0−91.0]	17.0 (9.8) [4.0−91.0]	
Vitamin B12, pmol/L	n = 604	n = 166	0.11
Mean (SD)	381 (495)	381 (327)	
Median (IQR) [Min-max]	298 (152) [92−4427]	318 (144) [92−3319]	

a Benjamini and Hochberg corrected p-value.

b Vitamin D is deseasonalized.

Non-included participants from the analyses were older, more often women, tended to have more cardiovascular disease and hypertension (Table 1).

Regarding vitamin status, the median (IQR) of our sample were 1.89 μmol/L (0.67) for vitamin A, 35.9 μmol/L (13.5) for vitamin E, 35.2 nmol/L (17.3) for vitamin D, 34.0 nmol/L (24.0) for vitamin B6, 17.0 nmol/L (110) for vitamin B9 and 298 pmol/L (152) for vitamin B12. Approximatively 75% of our sample is within the norms for vitamins A ([1.06–3.26 μmol/L]), E ([19.8–44.3 μmol/L]), B6 ([15−73 nmol/L]), B9 ([11−34 nmol/L]) and B12 ([100−600 pmol/L]). For vitamin D, nearly 25% had a sufficient status (>50 nmol/L).

The average follow-up time was 3.2 years (SD = 3.3, range = 0–10.8) with 16% participants having only one RNFL thickness measurement, 19% having two, 15% having three, 30% having four and 20% having five.

### 3.2 Multivariate analysis

Table 2 shows the linear mixed model analysis adjusted for age and sex (Model 1). At baseline, individuals with higher level of vitamin B6 had a thinner RNFL than individuals with a lower level (p = 0.03); vitamins A, E, D, B9 and B12 were not significantly associated with RNFL thickness. At baseline, after further adjustment for axial length, family history of glaucoma and alcohol consumption (for B vitamins) no circulating vitamin was associated with RNFL thickness (Model 2).

**Table 2:** Associations between circulating vitamins and retinal nerve fiber layer changes. Alienor Study 2009–2020.

		Baseline		Longitudinal change, μm/year	
	Participants/Eyes	*β*^a^ (95% CI)	*P* Value	*β*^a^(95% CI)	P Value
Model 1^b^					
Vitamin A	556/1072	0.18 (−0.97; 1.32)	0.76	−0.0001 (−0.10; 0.10)	0.99
Vitamin E	556/1072	0.02 (−1.21; 1.16)	0.98	0.12 (0.02; 0.22)	0.02
Vitamin D^c^	536/1037	0.92 (−0.23; 2.06)	0.12	0.15 (0.03; 0.26)	0.02
Vitamin B6	601/1161	−1.22 (−2.31; −0.13)	0.03	0.05 (−0.08; 0.18)	0.44
Vitamin B9	604/1167	−0.47 (−1.55; 0.60)	0.39	0.09 (−0.006; 0.18)	0.07
Vitamin B12	604/1167	−0.27 (−1.35; 0.81)	0.63	0.01 (−0.10; 0.12)	0.86
Model 2^d^					
Vitamin A	293/571	0.13 (−1.25; 1.51)	0.86	−0.02 (−0.12; 0.09)	0.78
Vitamin E	293/571	−0.21 (−1.60; 1.17)	0.77	0.13 (0.02; 0.24)	0.02
Vitamin D^c^	310/605	0.48 (−1.09; 2.06)	0.55	0.15 (0.02; 0.27)	0.02
Vitamin B6	317/619	−0.82 (−2.28; 0.65)	0.28	0.06 (−0.07; 0.19)	0.39
Vitamin B9	317/619	−0.30 (−1.61; 1.02)	0.66	0.09 (−0.008; 0.19)	0.07
Vitamin B12	317/619	−0.17 (−1.62; 1.28)	0.82	0.02 (−0.08; 0.13)	0.68
Model 3^e^					
Vitamin A	523/1009	0.12 (−1.08; 1.32)	0.84	0.007 (−0.10; 1.12)	0.89
Vitamin E	523/1009	0.20 (−1.00; 1.40)	0.74	0.14 (0.03; 0.25)	0.01
Vitamin D^c^	509/986	0.98 (−0.17; 2.13)	0.10	0.14 (0.02; 0.27)	0.02
Vitamin B6	533/1033	−1.30 (−2.60; 0.003)	0.05	0.07 (−0.06; 0.21)	0.30
Vitamin B9	535/1037	−0.41 (−1.56; 0.75)	0.49	0.11 (0.007; 0.21)	0.04
Vitamin B12	535/1037	−0.15 (−1.25; 0.94)	0.79	0.01 (−0.10; 0.12)	0.85

a For 1-SD increase in vitamin concentration.

b Model 1 is adjusted for age and sex.

c Vitamin D is deseasonalized.

d Model 2 is further adjusted for axial length and family history of glaucoma. Models for B vitamins are further adjusted for alcohol consumption.

e Model 3 is adjusted for age, sex, diabetes, hypercholesterolemia, hypertension and cardiovascular diseases. Models for B vitamins are further adjusted for alcohol consumption.

Individuals having higher concentrations of vitamin E and vitamin D had a slower RNFL thinning during the 10-years of follow-up. Indeed, a 1-standard deviation (SD) increase of vitamin E (10.8 μmol/L) and vitamin D (17.6 nmol/L) were associated with slower RNFL thinning by 0.12 μm/year (95% confidence interval (CI), 0.02−0.22, p = 0.02) and 0.15 μm/year (95% CI, 0.03−0.26, p = 0.02), respectively (Model 1). These associations remained similar after further adjustment in Model 2. No significant associations were observed for vitamins A, B6, B9 and B12 with RNFL longitudinal changes in these models.

The associations of vitamin E and vitamin D with RNFL thickness changes were also similar after adjustment for age, sex, diabetes, hypercholesterolemia, hypertension and cardiovascular diseases (Model 3). In model 3, individuals having higher concentrations of vitamin B9 had a slower RNFL thinning during the 10-years of follow-up. A 1-standard deviation (SD) increase of vitamin B9 (11 μmol/L) was associated with slower RNFL thinning by 0.11 μm/year (95% CI: 0.007−0.21, p = 0.04).

### 3.3 Sensitivity analysis

The associations of vitamin E, D and B9 with RNFL thickness were also similar in sensitivity analyses after adjustments on comorbidities (Model 3), after restriction to participants without glaucoma at baseline (n = 134), results, are display in Table 3. The associations of vitamin D with RNFL thickness were also similar in sensitivity analyses after exclusion of participants taking vitamin D supplements, results are display in Table 4.

**Table 3:** Associations between circulating vitamins and retinal nerve fiber layer changes among participants without glaucoma at baseline. Alienor Study 2009–2020.

		Baseline		Longitudinal change, μm/year	
	Participants/Eyes	*β*^a^ (95% CI)	*P* Value	*β*^a^ (95% CI)	P Value
Model 3^b^					
Vitamin A	479/923	0.48 (−0.70; 0.65)	0.43	0.01 (−0.10; 0.12)	0.83
Vitamin E	479/923	0.03 (−1.12; 1.18)	0.96	0.14 (0.03; 0.25)	0.01
Vitamin D^c^	474/918	0.80 (−0.31; 1.90)	0.16	0.17 (0.04; 0.30)	0.01
Vitamin B6	517/999	−1.30 (−2.40; 0.21)	0.02	0.06 (−0.08; 0.20)	0.38
Vitamin B9	494/957	−0.43 (−1.52; 0.65)	0.44	0.11 (0.006; 0.21)	0.04
Vitamin B12	494/957	0.22 (−0.88; 1.31)	0.70	0.008 (−0.11; 0.12)	0.90

a For 1-SD increase in vitamin concentration.

b Model 3 is adjusted for age, sex, diabetes, hypercholesterolemia, hypertension and cardiovascular diseases. Models for B vitamins are further adjusted for alcohol consumption.

c Vitamin D is deseasonalized.

**Table 4:** Associations between circulating vitamin D and retinal nerve fiber layer changes among participants without vitamin D supplement at baseline. Alienor Study 2009–2020.

		Baseline		Longitudinal change, μm/year	
	Participants/Eyes	*β*^a^ (95% CI)	*P* Value	*β*^a^ (95% CI)	P Value
Model 1^b^					
Vitamin D^c^	509/985	0.58 (−0.91; 2.07)	0.44	0.16 (0.04; 0.28)	0.01
Model 2^d^					
Vitamin D^c^	293/572	0.20 (−1.53; 1.94)	0.82	0.15 (0.03; 0.28)	0.02
Model 3^e^					
Vitamin D^c^	474/918	0.59 (−0.97; 2.09)	0.48	0.16 (0.04; 0.28)	0.01

a For 1-SD increase in vitamin D concentration.

b Model 1 is adjusted for age and sex.

c Vitamin D is deseasonalized.

d Model 2 is further adjusted for axial length and family history of glaucoma.

e Model 3 is adjusted for age, sex, diabetes, hypercholesterolemia, hypertension and cardiovascular diseases.

## 4 Discussion

This study adds new understanding of the potential protective effect of vitamins against retinal neurodegeneration by documenting the associations between circulating vitamins and longitudinal RNFL changes over 10-years in a cohort of French older adults. We evidenced that individuals with higher concentrations of plasma vitamin E, plasma vitamin D and serum vitamin B9 had a slower RNFL thinning over time. Vitamins A, B6 and B12 were not significantly associated with RNFL thinning in our cohort. This study also highlights that circulating status of vitamins A, E, D, B6, B9 and B12 were not associated with RNFL thickness at baseline.

To our knowledge, this is the first longitudinal study on the associations between circulating vitamins and RNFL thickness. Seven studies have reported associations between blood levels of vitamin E and glaucoma. Two case-controls studies reported lower plasma level of vitamin E in individuals with primary open angle glaucoma (POAG) [34] or in normal tension glaucoma (NTG) [35] while two other found increased serum vitamin E levels in glaucoma patients [36,37]. Three other studies found no difference in plasma levels of vitamin E between glaucoma patients and controls [[38], [39], [40]]. Concerning aqueous humor, lower levels of vitamin E have been reported in glaucoma patients [41]. Studies on the dietary intake of vitamin E and its association with glaucoma revealed no significant associations [10]. Oxidative stress reflects an imbalance between the production of reactive oxygen species (ROS) and the ability of cells to rapidly detoxify reactive intermediates or repair the resulting damage. Significant evidence has shown that oxidative stress plays a role in RGC death in glaucoma. Vitamin E has been shown to ameliorate N-methyl-D-aspartate (NMDA)-induced RGCs death, also vitamin E acts as a scavenger of peroxyl radicals [42,43].

A recent systematic review, based on cross-sectional and case-control studies, highlighted a lower vitamin D concentration in glaucoma patients compared to control group [44]. Vitamin D has anti-inflammatory properties by reducing the production of pro-inflammatory agents such as interleukins and inhibit T and B-lymphocytes [45,46]. Vitamin D is also known to be involved in several pathways critical to brain health -including neurotransmission, neuroprotection, and modulation of immune response-, pathways shared between retinal and brain structures [28,47]. Moreover, RNFL thinning was associated with cognitive performance and risk of dementia suggesting that neurodegeneration occurs simultaneously in the brain and the retina [48].

We also report that serum vitamin B9 could be associated with a slower RNFL thinning over time. Our result expand a previous case control study reporting a significant peripapillary RNFL thinning in patients with vitamin B9 deficiency (<7 nmol/L) [49]. Regarding glaucoma, some studies reported lower level of serum vitamin B9 in pseudoexfoliation glaucoma cases [18,50] while other studies found non-significant associations with POAG [17,[51], [52], [53]]. These studies are cross-sectional or case-control mainly based on a small number of participants. The biological mechanisms underlying the beneficial effect of folate on the retina are not well understood. One plausible explanation could be the potential role of vitamin B9 in DNA methylation processes. This nutrient plays a key role in one‐carbon metabolism and their deficiency could significantly reduce DNA methylation, leading to epigenome dependent changes in the expression of disease‐related factors [54,55]. Dysregulation in one‐carbon metabolism are linked to many neurodegenerative and age‐related diseases [56].

Also, the folate and methionine cycles are interconnected and folate deficiency could have deleterious effects on cells by allowing homocysteine accumulation [55]. Elevated levels of homocysteine have been shown to contribute to vascular damage [57] and oxidative stress [58]. Many age‐related diseases, including vascular diseases such as vascular occlusion or optic neuropathies as POAG [59] and pseudoexfoliation glaucoma [50], have been linked to higher homocysteine levels.

Finally, RNFL thinning in individuals with lower serum vitamin B9 might also be explained by impaired axon transport due to decrease oxidative phosphorylation and demyelination. Indeed, impairment of oxidative phosphorylation in mitochondria plays an important role in the pathophysiology of optic neuropathy. Oxidative phosphorylation in mitochondria ensures the production of adenosine triphosphate (ATP), and electrons transfer to oxygen. Vitamin B9 is particularly important for oxidative phosphorylation. Its deficiency leads to reduced ATP production in cells including cellular mitochondria. In demyelinated neurons, long axons are more attenuated, showing that the optic nerve is more sensitive to reduced ATP production [60,61].

The present study reports no cross-sectional association between RNFL thickness and circulating vitamin E, D and B9 while longitudinal association showed significant associations suggesting that vitamin E, D and B9 may be more involved in neurodegenerative process than associated with inter-individual structural differences in retinal morphology.

In our study circulating vitamins A, B6 and B12 were not associated with RNFL thickness at baseline neither with longitudinal changes. Regarding vitamin A, results from previous studies are divergent: two studies have reported lower serum vitamin A levels in NTG individuals compared to OAG individuals [14,36] while another had reported high levels in glaucoma patients [37], and three studies did not find any statistically significant associations [35,38,39]. Regarding dietary data, a meta-analysis of seven studies suggested that a higher intake of vitamin A is associated with a 37% decreased risk of glaucoma [10]. A meta-analysis reported no associations between serum vitamin B6 and B12 with glaucoma [62,63].

Our study presents some limitations. First, our sample size may seem small but compared to previous studies this is one of the largest. In our study sample, 16% of individuals had only one RNFL measurement available. This could lead to a limited statistical power and imprecision in the estimates. However, according to the literature, linear mixed models provide unbiased estimates when the missing data are MAR (missing at random) in case of non-exclusion of participants with only one measurement [64]. In observational studies, residual confounding is always a concern, and the potential beneficial effect of circulating vitamins might be explained by other factors. In the present study, results were similar in the parsimonious model (age and sex-adjusted) and the main model, suggesting that our results are not highly confounded. In secondary analyses, we further adjusted for comorbidities such as diabetes, serum cholesterol, hypertension and cardiovascular diseases and estimations were similar. In our study, selection bias cannot be completely dismissed, as included and non-included participants differed for some characteristics. However, this selection bias is limited, as they differed only regarding age, sex cardiovascular diseases and hypertension. Other limitations of our study include a single measurement of blood vitamins that does not allow us to measure changes over time and could only reflect recent nutritional status (previous months for vitamins D, B6 and B9; previous weeks for vitamin C and previous days for vitamins A, E and B12). Some of the biomarkers used in this study have relatively short half-lives (e.g. vitamin A≈12 days, vitamin E≈1 day and vitamin B12≈6 days), reflecting the subjects' recent nutritional status; and others have longer half-lives, reflecting longer-term nutritional status assessment (vitamin C≈20 days, vitamin B6≈30 days, vitamin D≈40 days, and, vitamin B9≈100 days). Thus, we measured serum vitamin B9 rather than red blood cell folate, considered as a better marker of chronic vitamin B9 deficiency. Regarding vitamin B3, a nicotinamide supplement (a derivative of vitamin B3) was associated with functional improvement in glaucoma patients in two small randomized trials [[65], [66], [67]]. Potential associations between vitamin B3 and changes in RNFL thickness are a hot topic. Unfortunately, in our study, vitamin B3 could not be measured, as it must be measured from whole blood samples, which are not available in our biobank. Furthermore, the assessment of exposure was, performed about nine years before the first ophthalmological examination. However, differential misclassification of circulating vitamins according to RNFL thinning seems unlikely, and thus misclassification of vitamin exposure would tend to bias the associations with RNFL toward the null.

Strengths of this unique study include its prospective design and long‐term follow‐up. Precise and repeated measurements of RNFL thickness were performed using SD-OCT with eye-tracking system allowing for high reproducibility and carried out by trained professionals according to standardized procedures. We used mixed linear models to take into account inter-individual and intra-individual variations over time, while considering intra-individual and intra-eye correlation and allowed to study the trajectories of RNFL thickness. The construction of different fitting models and a sensitivity analysis excluding participants with glaucoma at baseline argue in favor of the robustness of the obtained results. To our knowledge, the Alienor study is the first population-based cohort to allow studying the changes in RNFL thickness over time with 10 years of follow-up and multiple measures. Another strength of our study is the collection of biomarkers rather than dietary intakes to measure nutritional status. Biomarkers are more objectives and reproducible measurements of dietary status, limiting dietary assessment bias. Biomarkers also have the advantage to take into account the bioavailability. Our findings are likely generalizable to an older European Caucasian population and could also be relevant for other high-income countries.

## 5 Conclusion

In conclusion, this study suggests that high levels of vitamins E, D and B9 are associated with a slower RNFL thickness on SD-OCT over time, suggesting that these vitamins may contribute to the neuroprotection of the retina. Replication in other cohort studies would strengthen the present results.

## Funding

Alienor study is funded by Théa Pharma, Fondation Voir et Entendre, Agence Nationale de la Recherche (ANR 2010-PRSP-011 VISA), French Ministry of Health (PHRC 12_157, ECLAIR), CFSR Recherche (Club Français des Spécialistes de la Rétine) and 10.13039/501100001115CNSA (Caisse Nationale pour la Solidarité et l’Autonomie). Laboratoires Théa participated in the design of the Alienor study, but none of the sponsors participated in the collection, management, statistical analysis and interpretation of the data, or in the preparation, review or approval of the present manuscript.

## Ethical standards

The authors declare that the study procedures comply with current ethical standards for research involving human participants in France. This research was approved by the Ethical committee of Bordeaux (Comité de Protection des Personnes Sud-Ouest et Outre-Mer III) in May 2006. All participants provided written informed consent in accordance with the Declaration of Helsinki to participate in the study.

## Data statement

The dataset presented in this article are not readily available because of ethical and legal restrictions. Requests to access the dataset should be directed to the Steering Committee of the Alienor study (contact corresponding author: benedicte.merle@u-bordeaux.fr).

## Conflicts of interest

Bénédicte MJ Merle: outside the submitted work: consultant for Laboratoires Théa; grant from AXA.

Cédric Schweitzer: outside the submitted work: consultant Alcon, Abbvie, Bausch & Lomb, Glaukos, Horus, Johnson &Johnson, Nicox, Théa.

Marie-Bénédicte Rougier: outside the submitted work: board membership for Horus and Abbvie, lectures for Horus and Abbvie.

Audrey Cougnard-Grégoire: none.

Laure Gayraud: none.

Marie-Noëlle Delyfer: outside the submitted work: board membership for Abbvie, Bayer, Novartis, Roche and Thea, consultant for Abbvie, Bayer, Novartis and Roche.

Jean-François Korobelnik: outside the submitted work: consultant for Abbvie, Apellis, Bayer, Janssen, Thea, Carl Zeiss Meditec and Roche.

Cécile Delcourt: work under consideration for publication: grant from Laboratoires Théa. Outside the submitted work: consultant for Allergan Chauvin-Bausch + Lomb, Laboratoires Théa, Novartis; grant from Fondation de France; lectures from Apellis, Patent W020210589914A1.
